# Emotional enhancement of memory: how norepinephrine enables synaptic plasticity

**DOI:** 10.1186/1756-6606-3-15

**Published:** 2010-05-13

**Authors:** Keith Tully, Vadim Y Bolshakov

**Affiliations:** 1 Department of Psychiatry, McLean Hospital, Harvard Medical School, 115 Mill Street, Belmont, Massachusetts 02478, USA

## Abstract

Changes in synaptic strength are believed to underlie learning and memory. We explore the idea that norepinephrine is an essential modulator of memory through its ability to regulate synaptic mechanisms. Emotional arousal leads to activation of the locus coeruleus with the subsequent release of norepineprine in the brain, resulting in the enhancement of memory. Norepinephrine activates both pre- and post-synaptic adrenergic receptors at central synapses with different functional outcomes, depending on the expression pattern of these receptors in specific neural circuitries underlying distinct behavioral processes. We review the evidence for noradrenergic modulation of synaptic plasticity with consideration of how this may contribute to the mechanisms of learning and memory.

## Introduction

Specific cells and synapses within implicated neural circuits are recruited during learning, such that memory allocation is not random but rather is regulated by precise mechanisms which define where and how information is stored within the neural network [1]. Nearly four decades ago, Seymour Kety suggested that emotionally arousing experiences may be associated with activation of the locus coeruleus, sending adrenergic projections to different regions of the brain (such as hippocampus, cortex and cerebellum) [2]. Moreover, he proposed that activation of β-adrenoreceptors by released norepinephrine (NE) could result in facilitation of synaptic transmission through the mechanism involving increases in the intracellular cAMP concentration and new protein synthesis, thus contributing to the memory acquisition and maintenance. It is currently hypothesized that synaptic plasticity, specifically long-term potentiation (LTP), in the neural circuits of learned behaviors could provide a cellular substrate of memory storage [3]. Consistent with Kety's proposal, it has been demonstrated recently that direct activation of the locus coeruleus initiated protein synthesis-dependent LTP at the perforant path input to the dentate gyrus in awake rats [4]. At the behavioral level, there is overwhelming evidence that emotionally-charged events often lead to the creation of vivid long-lasting memories [5,6], in part due to a surge of norepinephrine and subsequent stimulation of adrenergic receptors in the nervous system [7,8], and, as a result, improved memory consolidation [6]. Unexpectedly, recent studies of the human subjects indicate that although emotionally-charged events are remembered better than emotionally neutral experiences, emotion may enhance the subjective sense of recollection more than memory accuracy [9].

The results of numerous previous experiments implicate the amygdala in acquisition and retention of memory for emotionally charged events [reviewed in [10-12]]. Thus synaptic enhancements in the conditioned stimulus (CS) pathways to the lateral nucleus of the amygdala were shown to contribute in the acquisition of fear memory to the acoustic CS during auditory fear conditioning [13-17]. It has been demonstrated also that the basolateral amygdala can regulate consolidation of memories in other regions of the brain [6,18]. The contribution of the amygdala to modulating memory consolidation critically depends on activation of β-adrenoreceptors in the BLA [19-21]. According to the emotional tagging concept, activation of the amygdala during emotionally arousing events could mark the experience as important and aid in enhancing synaptic plasticity in other regions of the brain [22]. Consistent with this notion, it has been shown previously that the actions of NE in the BLA promote the induction of LTP [23] and the expression of Arc protein, implicated in mechanisms of synaptic plasticity and memory formation, in the hippocampus [24]. On the other hand, plasticity in the auditory thalamus (specifically in the medial division of the medial geniculate nucleus and posterior intralaminar nucleus), prior to projections to the LA, plays an essential role in auditory fear conditioning [25,26]. This supports the notion that plasticity in multiple regions of the brain may contribute to the formation of fear memory [26].

Recent reviews have examined the role of the noradrenergic system in emotional memory [27], the influence of norepinephrine on fear circuitry [28], and the function of norepinephrine system in general [29]. Learning to recognize important cues in our environment with emotional salience, such as danger or altruistic social interactions, is an essential survival mechanism. Thus evolution has shaped our nervous system to robustly remember cues that elicit emotion. While some emotional responses are hard-wired into the brain's circuitry, many of them are learned through experience [10]. How do we remember emotionally charged events so well, and what does it tell us about the mechanisms of memory storage in the brain? Most of our experiences and information detected by our senses are not remembered. How does our brain know what events are important enough to be preserved for long-term storage? One important clue comes from the fact that the transition of the behavioral experiences into memory likely arises from changes in the efficiency of synaptic transmission in corresponding neuronal pathways [30-32,15]. In this review we will consider at the level of changes in synaptic function how creation of long-lasting memories during emotional arousal might be linked to a surge of norepinephrine in specific neural circuits.

## Mechanisms of NE production and routes of its delivery in the brain

**Figure 1:**
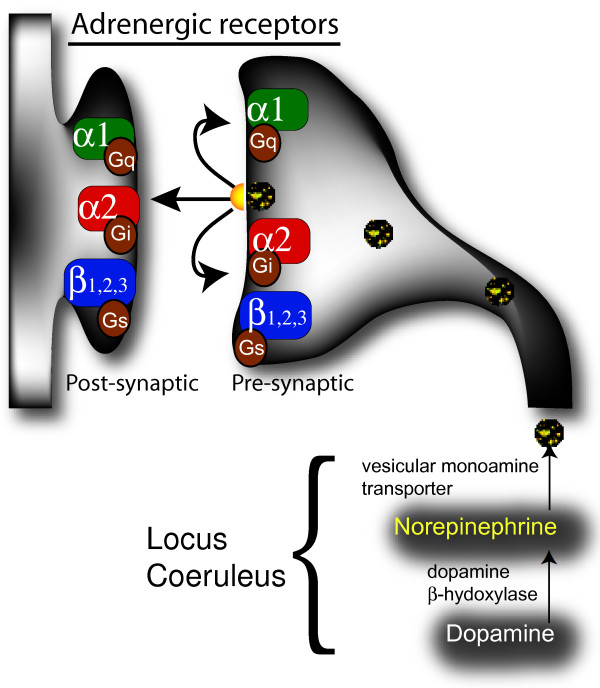
**Norepinephrine is synthesized from dopamine by dopamine β-hydroxylase in neurons of the locus coeruleus**. Before the final β-oxidation, norepinephrine is transported into synaptic vesicles by a vesicual monoamine transporter. The vesicles are then transported along the axons comprising the noradrenergic bundle to release sites. At the synapse norepinephrine is released into the synaptic cleft where it binds to various pre- and post-synaptic adrenergic receptors which subsequently activate distinct G protein coupled signal cascades.

Norepinephrine, also called noradrenaline, is a catecholamine produced by dopamine β-hydroxylase [33] which is released either as a hormone from the adrenal medulla into the blood or as a neurotransmitter in the brain. Norepinephrine in the brain is synthesized primarily in neurons in the locus coeruleus and to a lesser extent in the lateral tegmental field [29]. Within these neurons norepinephrine is transported by vesicular monoamine transporters into synaptic vesicles and carried along the axons composing the noradrenergic bundle to the sites of release [34] (Figure 1). These neurons send projections throughout the brain where norepinephrine performs its action upon release and binding to the G protein-coupled adrenergic receptors. It is followed by degradation of norepinephrine and/or its reuptake. There are two classes of adrenergic receptors, α and β, with each of them divided into several subtypes [35]. The subtypes of α receptors include G_q_-coupled α_1_receptors and G_i_-coupled α_2 _receptors. Activation of three different subtypes of β-receptors (β1, β2, β3), linked to G_s _proteins, results in a rise in the intracellular cyclic AMP concentration and subsequent PKA activation.

The locus coeruleus is a nucleus composed of mostly medium-size neurons located within the dorsal wall of the rostral pons in the lateral floor of the fourth ventricle that serves as the principal site for synthesis of norepinephrine in the brain [36]. The projections of the locus coeruleus spread throughout the central nervous system, with heavy innervation of the amygdala, brain stem, spinal cord, cerebellum, hypothalamus, thalamic relay nuclei, cingulate gyrus, hippocampus, striatum, basal telencephalon, and the cortex [37]. The action of norepinephrine in each neuroanatomical region is determined by the expression patterns of the various adrenergic receptor subtypes.

## The role of NE in memory

The mechanisms of memory could be best understood with the systematic application of associative learning paradigms. Short-term memory is normally defined and measured by retrieval tests that occur within the first few hours following the acquisition event, while long-term memory is typically measured using retrieval tests that occur at later times, following many hours or even days after the memory was acquired [3]. Numerous animal studies repeatedly demonstrated the role of adrenergic system in consolidation of memory for emotionally significant experiences [reviewed in refs. [6] and [38]]. In experiments using in vivo microdialysis and high-performance liquid chromatography (HPLC), NE release in the rat amygdala was detected in response to footshock stimulation which is usually used in inhibitory avoidance training paradigm [39]. NE levels were increased to approximately 75% above a basal concentration. Immobilization and tail pinch in rats were also shown to increase extracellular concentration of norepinephrine in the lateral and basolateral amygdala, as measured by microdialysis [40], leading to activation of adrenoreceptors present throughout the amygdala complex [41,42]. These findings were consistent with the notion that NE released by arousing stimulation could be involved in the functional regulation of neural circuits in the amygdala. Moreover, the amount of NE released in the amygdala during inhibitory avoidance training correlated strongly with 24-h retention performance [43]. Infusions of the β-receptor antagonist into the amygdala following inhibitory avoidance training resulted in amnesia in rats [44], while intra-amygdala injections of β-adrenergic agonists enhanced memory consolidation [19,45].

The acquisition of conditioned fear memory could also be modulated by pretraining manipulations of NE concentration in the amygdala [46]. Rats treated systemically with the GABA_A _receptor antagonist picrotoxin, used in the memory-enhancing doses, showed a substantial increase in levels of NE in the amygdala, while systemic injections of the memory impairing doses of the GABA_A _receptor agonist muscimol resulted in decreased levels of NE [47,6]. These findings supported the view that drugs that are capable of modulating emotional memory, such as GABAergic agonists and antagonists, may do so by controlling the level of NE within the amygdala [18,47]. Thus animal experiments have generally shown that injecting norepinephrine to various brain regions at times when memories are encoded or shortly after the behavioral training could enhance memory performance [27,48]. Conversely, blocking adrenergic receptors (such as β-type) could have a decremental effect on memory [6,49] and prevent the increase of memory performance during concurrent injections of the agonist [50]. According to a relatively recently proposed hypothesis, memory reconsolidation could occur when memory is retrieved and enters the labile state again, requiring additional protein synthesis for its transition into long-term memory [51,52]. It appears that the process of reconsolidation could also be modulated through activation of adrenoreceptors [53]. Based on cumulative evidence, release of NE in the amygdala is essential for encoding and retention of memories for the emotionally significant events.

Surprisingly, genetically-modified mice, in which the dopamine β-hydroxylase gene was ablated, did not exhibit any deficits in long-term fear memory following single-trial fear conditioning [54]. This was an unexpected finding because NE is not produced in these mice. It might be interesting to determine whether developmental compensations in dopamine β-hydroxylase knockout mice might be responsible for the lack of changes in conditioned fear memory in the absence of NE release during behavioral training. Moreover, postraining systemic or intra-amygdala administration of the β-antagonist propranolol had no effect on consolidation of fear memory in rats [55]. It is possible, however, that the concentration of propranolol in this study was too low to prevent completely activation of β-adrenoreceptor by endogenous NE, as the ability of amygala-injected propranolol to block consolidation of aversive memories was previously demonstrated [44].

The human subject studies provided further evidence that emotional arousal facilitates memory consolidation in a way that can be blocked by the β-adrenergic receptor antagonists, e.g. propranolol [6,38,56]. Thus, the blockade of β-adrenoreceptors impairs memory of an emotionally arousing story but does not affect memory of a closely matched emotionally neutral story [57]. In this classic study, the subjects who received propranolol remembered the emotional story no better than a neutral story. More recently, Segal and Cahill using an adrenergic biomarker showed that adrenergic activation relates selectively to memory for emotional events in humans [58]. One week after viewing a series of mixed emotional and neutral images a surprise recall test showed endogenous noradrenergic activation immediately after versus before slide viewing that correlated with the percentage of emotional pictures recalled. Functional magnetic resonance imaging has shown that encoding of emotional stimuli increases human amygdala responses, an effect that is blocked by administration of a β-adrenergic antagonist [59-61]. Interestingly, it has been demonstrated recently that emotional arousal could enhance the subjective sense of recollection enhancing confidence in the accuracy of the memory [9]. In these experiments, brain activity associated with remembering emotional and neutral photos was measured in combination with behavioral testing. In other words, emotionally arousing events may boost the feeling of remembering without enhancing the objective accuracy of memory [62].

By modulating memory for potentially threatening or harmfulstimuli, norepinephrine may ensure adaptive behavioral responses when such stimuli are encountered in the future.

## NE-mediated regulation of synaptic plasticity in the amygdala

Recent studies provide evidence that memory of fear could be acquired and, perhaps, retained through the mechanisms of LTP in the CS pathways [15,16]. The ability of glutamatergic synapses in fear conditioning circuits to undergo LTP is tightly controlled by several neuromodulators, such as gastrin-releasing peptide [63], vesicular Zn^2+^[64], or dopamine [65]. It has been demonstrated recently that stathmin, a phosphoprotein enriched in the amygdala and in the auditory CS and US areas, can control fear memory by regulating the susceptibility of cortico-amygdala and thalamo-amygdala synapses to LTP [66]. Could synaptic plasticity in the circuits underlying fear conditioning also be modulated by NE? A persistent late phase of LTP (L-LTP) in cortical and thalamic inputs to the LA, which is induced by repeated high frequency trains of presynaptic stimulation and is dependent on new protein synthesis, was found to be mediated by activation of β adrenoreceptors [67]. Conversely, activation of α2-adrenoreceptors impaired the induction of LTP at projections from the LA to the basal nucleus of the amygdala [68]. We have recently started exploring synaptic mechanisms by which norepinephrine might modulate plasticity in the CS pathways [69], linked to the acquisition of conditioned fear memory to auditory stimulation [10,12]. Although we found that norepinephrine had no direct effect on baseline glutamatergic synaptic transmission in thalamo-amygdala projections, by which the auditory CS information is conveyed to the LA from the auditory thalamus, the number of spikes triggered by depolarizing current injections in amygdalar neurons was increased in the presence of norepinephrine. This effect on neuronal firing properties is consistent with the observation that norepinephrine can inhibit slow after-hyperpolarization that follows trains of action potentials [70] and hyperpolarization-activated cation currents [71]. To examine the role of norepinephrine in modulation of synaptic plasticity in thalamo-amygala pathway, we paired stimulation of thalamic afferents with postsynaptic action potentials induced in a recorded neuron with a short delay after the presynaptic stimulus was delivered. This experimental protocol induced LTP in the presence of the GABA_A _receptor antagonist picrotoxin but not when GABAergic inhibition remained intact. While norepinephrine had no facilitatory effect on LTP induced in the presence of picrotoxin, the addition of norepinephrine to the external medium allowed the induction of LTP under conditions of intact inhibition when it otherwise would not have occurred. This led us to conclude that norepinephrine may enable the induction of LTP in thalamic input to the lateral amygdala by suppressing inhibitory GABAergic neurotransmission.

Consistent with the role of norepinephrine in the regulation of inhibitory drive, it has been found previously that norepinephrine reduced the frequency of spontaneous inhibitory postsynaptic currents in rat supraoptic neurons [72]. We further showed that norepinephrine reduced the frequency of spontaneous inhibitory postsynaptic currents in the LA and reduced the peak amplitude of disynaptic GABAergic inhibitory postsynaptic currents, strengthening the notion that NE may permit the induction of LTP by decreasing inhibition of principal neurons by local circuit interneurons. On the other hand, activation of α_2_-adrenoreceptors was shown previously to result in inhibition of excitatory postsynaptic responses in inputs to neurons in the basal nucleus, while activation of β-adrenoreceptors led to the strengthening of glutamatergic neurotransmission at the same synapses [73]. Consistent with these earlier findings, we found that application of norepinephrine in the presence of the α_2 _antagonist induced potentiation of thalamo-amygdala synaptic responses, an effect that was reversed by the β-adrenoreceptor antagonist. When norepinephrine was applied in the presence of the β-adrenoreceptor antagonist, the synaptic response was reduced, an effect that was partially reduced by the α_2 _antagonist. Thus, the oppositely directed effects on synaptic transmission during simultaneous activation of different adrenoreceptor subtypes are likely to be mutually exclusive under baseline conditions, but may allow for modulation during various behavioral processes in vivo. The ability of NE to gate LTP at glutamatergic synapses through the NE-induced suppression of GABAergic inhibition could, at least in part, explain a well-known ability of the GABAergic agonists and antagonist to suppress or promote memory mechanisms, respectively [18].

## Modulation of plasticity in the hippocampus

While the amygdala presents us with one of the clearest examples of how synaptic plasticity in a defined neural circuitry could control fear-related behavioral responses, the hippocampus is another important locus of the NE actions linked to the mechanisms of learning and memory. Using labeled norepinephrine, it was directly demonstrated that NE could be released in the hippocampus in an activity-dependent fashion. Thus, NMDA application was shown to induce the release of [^3^H] norepinephrine preaccumulated in slices from the hippocampus in both the dentate gyrus and the CA1 region [74]. A recent study has shown that norepinephrine-driven phosphorylation of GluR1 subunit may facilitate AMPA receptor trafficking to synaptic sites and LTP induction in the hippocampus [75]. The results of this work suggested that elevation of norepinephrine concentration during emotional arousal could lead to phosphorylation of GluR1, lowering the threshold for the experience-driven synaptic modifications and facilitating the formation of memories. Specifically, norepinephrine application resulted in phosphorylation of the GluR1 sites Ser845 and Ser831 which facilitated the synaptic delivery of GluR1 in CA1 neurons in the hippocampus. Moreover, norepinephrine could enhance contextual fear memory formation and LTP in wild-type mice but not in mice carrying mutations in the GluR1 phosphorylation sites. Norepinephrine application induced LTP when paired with a mild electrical stimulation, but not when it was applied alone. This indicates that synapses would undergo potentiation only if they are activated within a limited time window of emotional arousal associated with the surge of norepinephrine in the brain.

This recent study builds on an existing literature supporting a role of norepinephrine in the functioning of hippocampal circuitry. Thus, it was reported previously that norepinephrine could induce long lasting modifications of synaptic responses in the dentate gyrus in rats, associated with differential actions on distinct projections (medial vs. lateral perforant paths) arising in the entorhinal cortex [76]. The finding, that adrenergic antagonists could block amphetamine-induced increases in the dentate gyrus population spike in anesthetized rats and that an adrenergic agonist enhanced responses to NMDA, led to the conclusion that adrenergic receptors enhance reactivity of hippocampal cells to afferent stimulation [77]. It has been reported also that norepinephrine regulates synaptic plasticity in the CA1 area of young rats [78], causing a shift in the frequency-response relationship for long-term depression (LTD) induced with the low frequency theta-burst stimulation and LTP (observed at higher frequencies), in the β-adrenoreceptor-dependent fashion. Norepinephrine caused a shift toward potentiation, with the effect of norepinephrine being most prominent at intermediate frequencies, which induced no changes in control slices but strong LTP in the presence of norepinephrine. It has been found later that an administration of norepinephrine in the CA1 region in hippocampal slices prepared from more mature animals allowed for more robust LTP under the conditions when reduced LTP was normally observed [79]. The effects of norepinephrine were mimicked by the α_1_-adrenoreceptor agonist and were blocked by the α_1_-adrenoreceptor antagonist, leading to the conclusion that the actions of norepinephrine may shift during development. Activation of noradrenergic systems during emotional arousal may enhance memory formation by inhibiting protein phosphatases that normally oppose the induction of LTP, because protein phosphatase inhibitors mimicked the effects of β-adrenoreceptor activation that enabled the induction of LTP during long trains of stimulation at CA1 synapses [80]. In the experiments on 6-hydroxydopamine-treated rats, lacking norepinephrine, it was shown that LTP but not LTD was blocked at CA1 synapses, while application of norepinephrine restored LTP and blocked LTD [81]. This plasticity occurred through activation of β-adrenoreceptors and involved the cAMP/PKA pathway. Importantly, several studies directly demonstrated that activation of locus coeruleus neurons produces adrenoreceptor-mediated LTP-like synaptic enhancements in the dentate gyrus [4,82-84]. The release of NE in the hippocampus during emotionally-charged events could thus modulate the hippocampus-dependent forms of memory by controlling the induction of synaptic plasticity at corresponding synapses in hippocampal neuronal circuits.

## The role of second messengers in regulation of synaptic plasticity by NE

The better understanding of the mechanisms by which emotional arousal could enhance the brain's ability to store, retain, and subsequently recall information, would require a detailed characterization of the signaling pathways lying downstream of norepinephrine binding to adrenergic receptors. Norepinephrine activates various subtypes of adrenergic receptors, so it is not surprising that evidence is accumulating for the differential roles of signaling pathways tied to distinct receptor classes in synaptic plasticity. Thus, the extracellular signal-regulated MAP kinase (ERK) is activated by β-adrenoreceptors in somas and dendrites of CA1 pyramidal neurons in a PKA-dependent fashion [85]. It plays a regulatory role in the induction of certain forms of LTP at the Schaffer collateral-CA1 synapses. ERK was found to be required for the early phase of LTP elicited by brief presynaptic stimulation, as well as for LTP elicited by prolonged stimulation paired with β-adrenoreceptor activation in CA1 pyramidal neurons. In a later study, coactivation of β-adrenoreceptors and cholinoreceptors was found to enhance LTP at CA1 synapses through convergent, synergistic activation of mitogen-activated protein kinase [86]. In another work, synaptic stimulation, which was subthreshold for the induction of late phase LTP (L-LTP), triggered this form of plasticity when LTP-inducing stimulation was delivered in the presence of the β-adrenoreceptor agonist [87]. The induction of this form of LTP also required activation of ERK. Further research is clearly needed to decipher the signaling cascades initiated by the binding of norepinephrine to adrenergic receptors linked to the various G proteins, mediating activation of the specific signal transduction pathways and implicating either adenylyl-cyclase (through G_i_-coupled α_2 _receptors and G_s_-coupled β1, β2, and β3 receptors) or phospholipase C (through G_q_-coupled α_1_receptors). This knowledge would result in understanding of how second messengers may interact with the mechanisms of synaptic plasticity in the brain in the context of specific forms of learning and memory.

## The effects of NE in other neural circuits

As the noradrenergic bundle sends projections throughout the central nervous system, it is expected that norepinephrine would modulate synaptic plasticity in other neural circuits besides the hippocampus and amygdala. Consistent with this prediction, it has been demonstrated recently that activation of α_2 _adrenoreceptors by NE in Purkinje cells could control short-term and long-term associative plasticity at the parallel fiber synapses [88]. In the cerebellar circuitry, norepinephrine was affecting synaptic plasticity by decreasing the probability of release at the climbing fiber synapse, which in turn decreased the climbing fiber-evoked dendritic calcium signals. In addition, this study demonstrated that norepinephrine was acting presynaptically to decrease the probability of neurotransmitter release at climbing fibers but not at granule cell parallel fibers. The reduction in dendritic calcium elevation, associated with complex spikes, was independent of postsynaptic G protein signaling. Moreover, activation of α_2_-receptors interfered selectively with the induction of associative synaptic plasticity. Thus, noradrenergic modulation in this system could provide a mechanism for context-dependent modulation of associative plasticity and memory. In slices of the rat visual cortex, paired-pulse stimulation in the presence of NE resulted in a form of homosynaptic LTD [89]. This noradrenergic facilitation of LTD was blocked by the α_1 _receptor antagonist and mimicked by the α_1 _agonist. In the developing visual cortex in rats, LTP at inhibitory synapses could be induced by the high frequency stimulation when excitatory neurotransmission was blocked [90]. The induction of this form of LTP, mediated by presynaptic calcium entry, was facilitated by norepinephrine.

In several brain circuits, α_2_-adrenoreceptor-dependent inhibition of excitatory glutamatergic signaling was observed. Norepinephrine, acting at presynaptic α_2 _receptors, inhibited single fiber glutamatergic inputs from the nociceptive pontine parabrachial nucleus to the central amygdala [91]. This effect of NE, mediated by decreases in the number of active release sites, could potentially explain how norepinephrine can decrease pain sensation under stress. Activation of presynaptic α_2_-adrenoreceptors on inputs to sympathetic preganglionic neurons in slices of neonatal rat spinal cord also decreased glutamate release [92]. In these experiments, norepinephrine produced dose-dependent and reversible decreases in the amplitude of the excitatory postsynaptic response. Interestingly, the previous experiments, using a microdialysis technique to estimate the extracellular levels of norepinephrine and glutamate in the bed nucleus of the stria terminalis in rats, demonstrated that norepinephrine could exert α_2_-dependent inhibition over both its own and glutamate release [93]. At the calyx of Held synapses, NE was shown to increase the high frequency firing. This was associated with the initial suppression of glutamatergic excitatory postsynaptic currents through α_2_-dependent inhibition of calcium influx [94].

Taken together, these findings indicate, that while NE can produce a variety of the effects on synaptic and neuronal mechanisms in different regions of the brain, the expression patterns of different NE receptor subtypes in specific neural circuits could determine the resulting functional outcome.

## Modulation of GABAergic transmission by NE

Presently, there is substantial evidence that activation of adrenoreceptors by norepinephrine can modulate GABAergic inhibitory systems in the brain. Thus, activation of the locus coeruleus resulted in suppression of feedforward interneurons in rat dentate gyrus, thereby promoting conditions for the induction of synaptic plasticity [95]. In the LA, NE was inducing hyperpolarizing currents in local circuit interneurons leading to their decreased excitability, and, therefore, to decreased feedforward inhibition of principal neurons [69]. This facilitated the induction of LTP at thalamo-amygdala synapses. Additionally, there are multiple reports suggesting that norepinephrine could, in fact, enhance inhibition. Thus, NE excited medial septum and diagonal band of Broca GABAergic neurons [96]. NE was shown also to increase the frequency and the amplitude of GABAergic inhibitory postsynaptic currents in substantia gelatinosa [97]. In CA1 pyramidal neurons, norepinephrine increased action potential-dependent inhibitory postsynaptic currents by depolarizing surrounding inhibitory interneurons [98]. It was reported also that NE can potentiate the Purkinje cell responses to GABA due to triggering the signaling cascade involving G_s_-linked β-adrenoreceptors activating the cAMP-dependent pathway [99]. In the hypothalamic paraventricular nucleus, NE was shown to increase the frequency of spontaneous inhibitory synaptic current via postsynaptic α_1_-adrenoreceptors and decrease it through activation of α_2_-adrenoreceptors on GABAergic terminals [100]. As the susceptibility of central synapses to LTP is determined by the strength of GABAergic inhibition, NE can contribute to the behaviorally-induced plasticity in the brain through its ability to modulate inhibitory inputs to projection neurons.

## Conclusions

It is well established that emotional arousal modulates the formation of memory, and a substantial literature, of which only a fraction is cited in this review, points to a critical role for the release of norepinephrine in such modulation. The electrophysiological studies have begun to elucidate how norepinephrine could modulate both synaptic transmission and plasticity in specific neural circuits. Storing memories in the brain likely requires changes in the number, structure, and function of synapses [3]. Considerable progress has been made in relating the activity-dependent changes in synaptic strength to the mechanisms of learning and memory [101]. It appears that the mechanisms by which the release of norepinephrine during emotional arousal affects memories most likely involve modulation of synaptic plasticity in corresponding neural circuits. As investigative technologies, allowing manipulating the expression of specific proteins through genetic or epigenetic means, or techniques for delivery of pharmacological agents to the specific sites in the brain mature over the coming decade, the promise of therapeutic strategies for the treatment of a host of mental illnesses may be realized. It is critical that, in parallel, we understand in detail the mechanisms by which norepinephrine may alter memory processing through its interactions with the various pre- and post-synaptic adrenoreceptors in specific neural circuits.

## Abbreviations

AMPA: α-amino-3-hydroxyl-5-methyl-4-isoxazole-propionate; cAMP: Cyclic adenosine monophosphate; ERK: extracellular signal-regulated kinase;GABA: γ-aminobutyric acid; GluR1: AMPA receptor subunit; LTP: long-term potentiation; LTD: long-term depression; MAPK: mitogen-activated protein kinase; NMDA: N-methyl-D-aspartic acid; PKA: cAMP-dependent protein kinase

## Competing interests

The authors declare that they have no competing interests.

## Authors' contributions

K.T. and V.Y.B. wrote, read and approved the final manuscript
